# Immune Activation Efficacy of Indolicidin Is Enhanced upon Conjugation with Carbon Nanotubes and Gold Nanoparticles

**DOI:** 10.1371/journal.pone.0123905

**Published:** 2015-04-15

**Authors:** Abhinav Sur, Biswaranjan Pradhan, Arka Banerjee, Palok Aich

**Affiliations:** School of Biological Sciences, National Institute of Science Education and Research, Bhubaneswar, India; Second University of Naples, ITALY

## Abstract

Antibiotic resistance is concern of today's world. Search for alternative molecules, for treatment and immune stimulation, remains at the forefront. One such group of biomolecules with promise, along the line of immune stimulation or therapy, is host defense peptide (HDP). These molecules, however, are required at a higher dose to be effective which leads to high cost. To alleviate such problems, an aid can be used to achieve similar efficacy but at a smaller effective dose of the immune stimulant. We hypothesised that by conjugating HDPs with carbon nanotubes and/or gold nanoparticles, it would be possible to stimulate a protective immune response in host system at a lower dosage of HDP. In this report, we characterized, using biophysical methodologies, conjugation of Indolicidin, as a representative of HDP. We further established efficacy of peptide-nanomaterial conjugates in activating innate immunity and protecting against pathogen infection *in vitro* at a significantly small dose.

## Introduction

Due to the declining efficacy of existing antimicrobials, there is a need to generate new ones. A promising new approach for the treatment of infectious disease is through activation of innate immune system rather than direct attack on the microbe [1, 2]. This is anticipated to be of therapeutic and prophylactic significance as the effective treatment of a particular infection will be dependent upon the activation of appropriate aspects of innate immunity [3–7]. This strategy harnesses the natural power of immune responses and may minimize the likelihood of bacterial resistance as the attack is indirect, multi-faceted and evolutionarily successful. Innate immunity is mediated through a complex network of cellular and molecular systems that mediate a spectrum of biological activities which include: up-regulation of cytokines or chemokines and their receptors, recruitment of leukocytes to sites of infection, phagocytic cell activation, activation of extracellular killing mechanisms, stimulation of histamine release, angiogenesis, dendritic cell maturation and wound healing [1–3]. In search of molecules as alternative to antibiotcis, host defense peptides (HDPs) are promising ones. Although HDPs are noted for their antimicrobial activity, our interest is restricted to their immunomodulatory capabilities [8, 9]. It has been demonstrated that HDPs play important role in the innate immune system by constituting the first line of defense against pathogen assault [10, 11]. In this capacity, HDPs are among the leading candidates to serve as templates for the creation of novel antibiotics via induction of innate immune responses [12]^,^[13].

Despite all such potentials of HDPs, very little has been achieved clinically because of weak activity, nonspecific cytotoxicity and proteolysis of the molecules in general [14]. In order to alleviate these problems, a ‘dosage compensation’ module needs to be developed to enhance efficacy of these molecules. We hypothesized that using nanoparticles such as carbon nanotubes (CNTs) or gold nanoparticles (GNPs) efficacy of HDPs can be enhanced [15–17]. We selected indolicidin, as a representative cathelicidin HDP molecule for the current study, because it had been tested for various immune modulatory effects [18]. Indolicidin is a natural tridecapeptide cationic cathelicidin HDP containing five tryptophan (Trp) and two proline (Pro) residues [19]. Indolicidin is antibacterial, antifungal, antiparasitic, antiviral, immunemodulator and an inhibitor of aminoglycoside antibiotic resistance enzymes [10, 11, 20–22]. Here, we report (a) detailed characterization of conjugation of indolicidin with either CNT or GNP and (b) following conjugation of indolicidin with CNTs and GNPs, we were able to enhance efficacy of indolicidin *in vitro*. Our results revealed that we can maintain the activity of peptide at a significantly lower (1000-fold less) dosage.

Current approach is particularly important to counteract emergence of bacterial resistance to antibiotics, which has led to an increased urgency to explore alternative means of combating pathogenic assault. It has been shown that immunostimulatory molecules such as synthetic CpG oligonucleotides, anti-microbial peptides and host defense peptides can be beneficial in protecting against bacterial infection [18]. Host Defense Peptides (HDPs) have been shown to enhance immune responses and protect against infection by pathogens [10, 11]. The contrasting direct antimicrobial and immunomodulatory activities make these peptides valuable in the design of alternatively directed therapeutic agents and as tools in dissecting the variations in the mechanisms that underpin these diverse activities. In addition, their small size makes them potentially exciting prototypes for development as novel immunomodulatory drugs, especially because of their collective ability to enhance chemokine production, induce chemotaxis and block endotoxin responses. These properties make these peptides the potential to become an entirely new therapeutic approach against bacterial infections [6]. We further established anti-bacterial activity of conjugated indolicidin-CNT molecule.

## Materials and Methods

### Materials

Indolicidin (IR-13 GL Biochem P120313-HR187616), 1-ethyl-3-(3-dimethyl- aminopropyl) carbodiimide (EDC), N-hydroxysuccinimide (NHS), 2-(N-morfolino) ethanesulfonic acid (MES) pH = 5.0, Phosphate Buffer Saline (PBS), 2-mercaptoethanol, Dimethyl formamide (DMF), Dimethyl Sulfoxide (DMSO). COOH functionalized short multi-walled carbon nanotubes (SMWCNTs) purchased from CheapTubes, USA, COOH functionalized gold nanoparticles (3nm diameter) purchased from Nanocs, USA.

### Synthesis of CNT-Indolicidin and GNP-Indolicidin conjugates

**Fig 1:**
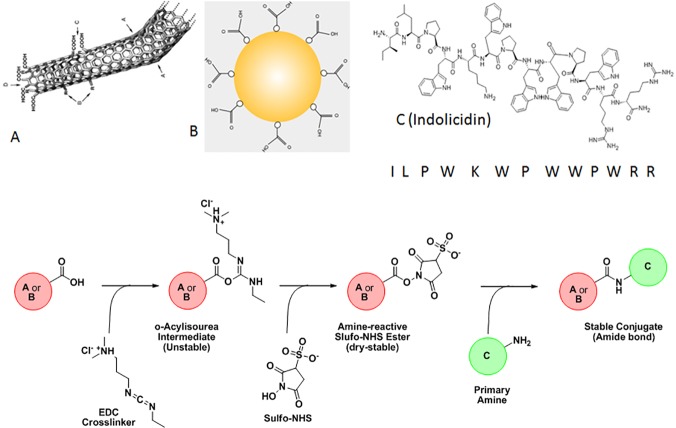
A schematic diagram of synthesis protocol of nanoparticle conjugation with indolicidin is shown. Letters 'A', 'B' and 'C' denote carboxylated CNT, carboxylated GNP and Indolicidin with primary amine.

Host defense peptide, Indolicidin, was conjugated to the nanomaterials (GNP and CNT) using EDC-NHS conjugation protocol[23]. 5 mg of indolicidin (IR-13 GL Biochem P120313-HR187616) was suspended in 25 μl of DMSO. The resulting solution was mixed properly followed by further addition of 975 μl of PBS to make a 5 mg/ml peptide solution. This solution was used as the stock peptide solution for our experiment. 400 μl of the 1 mg/ml CNT solution or the 12 nM GNP solution, prepared earlier was put in a clean and sterile microfuge tube. To the above solution, 600 μl of MES buffer (pH = 5.0) used as the appropriate activation buffer was added. This is because activation of the carboxyl groups on the nanotubes using EDC and NHS is most efficient at pH = 4.5–7.2. 5 μl of 0.4 M EDC and 50 μl of 0.1 M NHS was added respectively and the solution was incubated in dark for 45 minutes at room temperature. Once the activation reaction is complete, 1.4 μl of 2-mercaptoethanol was added to quench the effect of EDC. 960 μL of PB (pH = 7.2) was added to 1 ml of the activated solution. The solution was mixed by gentle pipetting. PBS is used as the conjugation buffer. Therefore, after adding 40 μl of the stock 5 mg/ml peptide solution, the resulting solution was mixed thoroughly and incubated in dark for 2 h at room temperature. In addition, free indolicidin was diluted to the similar extent for proper comparison to the conjugates. Spike was prepared by adding same concentrations of indolicidin to a solution of non-activated CNTs and GNPs. Free peptides were removed from the conjugate mixture using molecular weight cut-off spin columns (3 MWCO, Millipore, USA). Fig 1 depicts the basic algorithm of the synthesis scheme.

### Physical characterization of the indolicidin conjugates

Once the CNT and GNP adducts were synthesized, the conjugate needed to be validated. Therefore, the characterization of the conjugation reaction was conducted using the following methodologies:

#### UV-Vis Spectroscopy

UV-Vis spectrophotometry was conducted using NanoDrop2000 (Thermo-Scientific, USA) in the wavelength range of 200–550 nm.

#### Steady State Fluorescence Spectroscopy

Fluorimetric measurements were obtained using a PerkinElmer (LS55) instrument by exciting the samples at 280 nm and recording the emission spectra in the wavelength range of 290 nm to 600 nm using an excitation and emission slit width of 10 nm and a 1% attenuation of the emission signal in case of the samples corresponding to the CNT-peptide conjugation and at a 7 nm slit width for the GNP-peptide combinations for proper resolution of the spectra.

#### Time resolved florescence spectroscopy

Time-resolved fluorescence measurements were carried out using a time-correlated single-photon counting (TCSPC) spectrometer (Edinburgh, OB920). The samples were excited at 280 nm using picoseconds laser diode (EPL), and the signals were collected at magic angle (54.7°) using a Hamamatsu micro channel plate photomultiplier tube (R3809U-50). The lamp profile was recorded by scatterer (dilute Ludox solution in water) in place of the sample. The instrument response functions (FWHM) of our setup is ∼840 picoseconds for 280 nm. Decay is analyzed at 358 nm. Decay curves were analyzed by nonlinear least-squares iteration procedure using F900 decay analysis software. The qualities of the fit were judged by the chi square (χ2) values, and weighted deviations were obtained by curve fitting.

#### Fourier Transform Infrared (FTIR) Spectroscopy

FTIR was conducted using PerkinElmer Spectrum RX1 instrument. FTIR spectra measurements were obtained on purified lyophilized samples in the absorbance range from 4000 to 400 cm^-1^ by accumulating 20 scans with a spectral resolution of 1 cm^-1^. Absorbance spectra were corrected versus a spectrum of potassium bromide, obtained in the same instrumental conditions.

#### Binding Isotherm

Free nanoparticles after activation of their carboxyl groups, titrated against increasing concentrations of the peptide yielded a gradual increase in the absorbance and fluorescence intensities till it reached a saturation point. From the saturation binding curve, a corresponding binding isotherm for CNT-Indolicidin and GNP-Indolicidin conjugation was generated in order to calculate the affinity constants of the peptide for the two different nanomaterials and the stoichiometry of binding in each case. In addition, free non-activated nanoparticles were also titrated against increasing concentrations of indolicidin in order to understand the presence of any non-specific interactions between the free peptide and the functionalized nanoparticles. All analyses were done using GraphPad Prism 5.01 software, CA, USA.

#### Isothermal Calorimetry

Investigation of the peptides’ potential to interact with free activated nanoparticles was conducted utilizing the GE Healthcare MicroCal^TM^iTC_200_ Isothermal Calorimetry apparatus. The settings were with few exceptions, 40 injections of 1μl with a spacing of 300 seconds and an injection time of 5 seconds and baseline setting at 10μcal. Furthermore, a 2000 second delay was applied to get a steady baseline before injection of ligand. From the signal obtained, the thermodynamic parameters related to the binding of the peptide to the carboxyl groups on the nanomaterials were calculated.

#### Scanning Electron Microscopy

50 μl of aqueous dispersion of the conjugates and the free nanoparticles were spotted on a silicon chip and spin coated for 3 minutes at 1000 rpm under vacuum. The spin coated samples were baked on a dry bath for 1 minute before they were inserted into the instrument for image analysis. The samples on the chips were observed in electron microscopy with an accelerating voltage of 5.0–6.0 kV.

#### Animal Cell Culture

RAW264.7 murine macrophage cells and THP-1 human monocyte cell lines were obtained from ATCC (Manassas, VA). Both cell lines were maintained in RPMI-1640 (Himedia #AL008) supplemented with 4.5 gL^-1^ D-glucose, 25mM HEPES, 0.11 gL^-1^ sodium pyruvate, 1.5 gL^-1^ sodium bicarbonate, 2mM L-glutamine and 10% FBS along with 100 units ml^-1^ Gentamycin and 100 pg ml^-1^ Amphotericin-B. For proper comparison, THP1 primary monocytes were differentiated into adherent macrophages by treating them with phorbol-12-myristate-13-acetate or PMA. Following PMA treatment, THP-1 monocytes were incubated for 48 h before treatment with all the different conditions.

Around 2 million RAW264.7 cells and differentiated THP-1 cells were seeded into each well in a 6 well plate and were incubated at 37°C and 5% CO_2_ for 24 h to allow the cells to recover. The medium from each well was aspirated and 3 ml of fresh growth medium was added per each well. To the confluent cell layer, a standardized dose of free indolicidin (20 μg/ml) and a lower dose of indolicidin (0.02 μg/ml) conjugated to CNTs and GNPs were added, and further incubated for 6 h at 37°C. In addition, the cells were also treated with a lower dose of free peptide (0.02 μg/ml) for a relative comparison against the conjugates. The medium was then aspirated from each well and the cell layer was rinsed 3 times with PBS to remove any traces of unwanted treatment samples. About 0.3 ml of 0.1 M Trypsin-EDTA solution was added to each well to detach the cell layer from the plastic. Detached cells were dispersed in 1 ml of complete growth medium and gently pipetted out of the well. The cell suspension was transferred into a centrifuge tube and centrifuged at approximately 300 x g for 5 minutes. RNA was extracted from the cells using the Qiagen RNeasy Mini Kit as per the protocol described in the manual.

#### RNA Isolation

Total RNA was extracted from the nanoconjugatetreated and control cells using RNeasy mini kit (Qiagen # 74106) using the specified protocol. The concentration of extracted whole RNA was measured using Nanodrop 2000 instrument (Thermo Scientific). The value for A_260_/A_280_ and A_230_/A_280_ were noted to determine the quality of RNA. Quality of extracted RNA was also checked using Agilent 2100 Bio-analyzer Instrument, employing the standard protocol given in Agilent Bio-analyzer RNA 6000 nano kit (#5067–1511). The quality of total RNA was assessed by comparing the ratio of the area under the ribosomal peaks for 28S and 18S rRNA. RNA samples with a RIN value of 8.5 and more was considered of adequate quality and was used for further analyses.

Depending on the RNA quality and RNA concentrations, these RNA samples were used for complementary DNA synthesis. The cDNA samples thus obtained were used for further transcriptomic analysis of certain select innate immune genes.

#### cDNA Synthesis

Complementary DNA (cDNA) was synthesized by reverse transcribing total RNA for each condition in a 30 μL reaction containing the following: 14.5 μl of Herculase II RT-PCR 2X Master Mix, 200 ng of gene-specific reverse primer or oligo-dT primer, 5μl of AffinityScript RT-block and 3 μg of template RNA. RT reactions were first incubated at 25°C for 5 minutes to allow primer annealing followed by cDNA synthesis at 42°C for 45 minutes. Once cDNA is formed, the reactions were incubated at 95°C for 5 minutes to terminate the reaction. The completed first-strand cDNA synthesis reactions were placed on ice for immediate use in qRT-PCR reactions. The synthesized cDNA concentrations were quantified using the NanoDrop2000 spectrophotometer.

#### qRT-PCR

Gene expression studies were conducted on murine macrophage RAW264.7 cells to understand effects of free indolicidin and its conjugated and non-conjugated counterparts on the innate immune system *in vitro*. A preliminary transcriptomic analysis using qRT-PCR was conducted in all cases using GoTaq qPCR Kit Promega (#A6002) in order to generate a comparative expression profile of early select innate immune genes 6 h after treatment. The final 25 μl PCR reaction mixture comprised the following volumes and concentrations for a 96 well plate reaction: 9.4 μl of 2X GoTaq qPCR Master Mix, 12.6 μl of nuclease free water, 100 ng of template cDNA and 1 μMolarof each forward and reverse primers. The primers used for the preliminary transcriptomic analysis are tabulated in S1 Table. PCR amplification was performed in a programmable thermocycler (Stratagene 3500Mxp).

The PCR cycle consisted of a 30 second denaturation step at 94°C, 15 seconds annealing step at 60°C and 30 seconds elongation step at 72°C. Fluoroscence intensity was recorded after every elongation step. An initial 2-min denaturation step at 95°C as well as for HotStarTaq*Plus* activation. Real time PCR results were expressed as C_t_ (cycle threshold) values. This value corresponded to the cycle at which the fluorescence of the Sybrgreenprobe reached above the threshold or background fluorescence value. In quantifying gene expression, the mRNA levels of the gene of interest were divided by the mRNA levels of the housekeeping gene β-actin. This normalized for the variations in concentration and quality of mRNA among the samples. The final value reported was the ratio below:

$$Ratio={\left(1+E_{gene\_of\_\mathrm{int}erest}\right)}^{\Delta C_{tt}(control-treated)}/(1+E_{hoousekeeping\_gene)}{}^{\Delta C_{t}(normal-treated)}$$

Where, E is the primer efficiency determined by the qRT-PCR machine.

#### Bacterial Protection Assay

Around 0.5 million differentiated THP-1 cells were seeded into each well of a clean and sterile 12-well plate and incubated at 37°C for 24 h. Confluent cell layer was treated in triplicates with a standardized dose of free indolicidin (20 μg/ml) and a lower dose of indolicidin (0.02 μg/ml) conjugated to CNTs and not conjugated to CNT. In addition, the cells were also treated with a lower dose of free peptide (0.02 μg/ml) and free CNTs for proper comparison against the conjugates. After treatment, the plates were further incubated for 6 h at 37°C.

At a 6 h time point after treatment the THP-1 cells were infected with *Salmonella typhimurium* at a multiplicity of infection (MOI) of 10. The bacteria were grown to late log phase to ensure the expression of SP-1 genes so that both the invasive and phagocytic mechanisms can occur. After infection, THP-1 cell counts were recorded at 6 h, 12 h and 18 h time-points following Trypan blue staining. At each 6 h interval, the infected cells were collected by detaching them from the plastic using 0.1M Trypsin-EDTA solution and dispersing them in 0.5 ml of growth medium. The cellular suspension was further diluted and to the diluted suspension, 100 μL of Trypan blue stain (1% solution) was added. After vigorous mixing, 10 μL of the cell suspension was loaded onto a haemocytometer and the cell counts at that particular time point were recorded.

## Results

**Fig 2:**
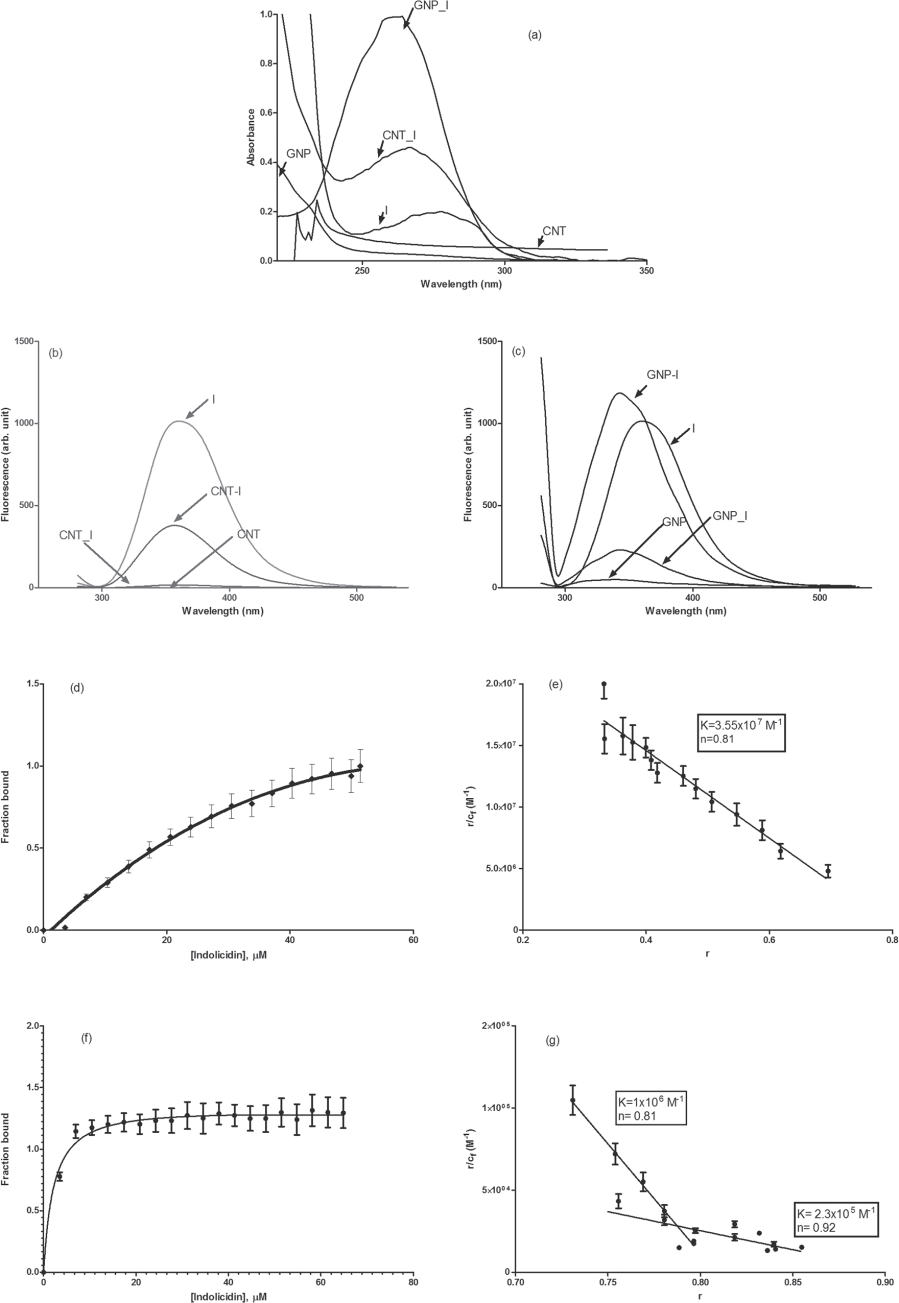
Absorbance and fluorescence spectra and binding isotherm. Representative (a) absorbance spectra of free indolicidin (I), free CNT, free GNP, CNT conjugated I (CNT_I) and GNP conjugated (GNP_I) indolicidin and fluorescence spectra of (b) free CNT, CNT-I, free I and free CNT spiked with indolicidin and of (c) free GNP, GNP-I, free I and free GNP spiked with indolicidin are shown. Spectra are labeled in the panels for easy identification. Binding isotherm of indolicidin with CNT and GNP. Fraction of indolicidin bound with (a) CNT and (c) GNP and Scatchard plot of binding of indolicidin with (b) CNT and (d) GNP are shown. Panels (b) and (d) also listed the values of association constant and stoichiometry of binding. Standard deviations of the data are shown as error bars in the figure.

**Fig 3:**
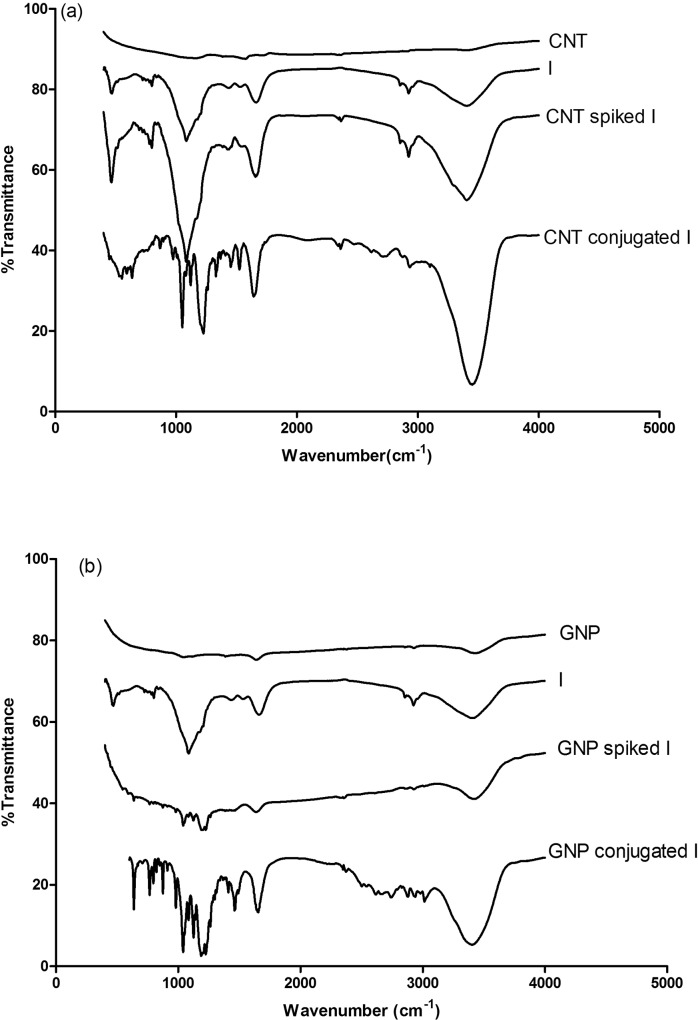
FTIR spectra. Representative fourier transformed infrared spectra of (a) free CNT, free Indolicidin (I), CNT spiked I (CNT-I) and CNT conjugated with I (CNT_I) and (b) free GNP, free I, GNP spiked I (GNP-I) and GNP conjugated with I (GNP_I) are shown. Each spectrum is labeled for easy identification.

**Fig 4:**
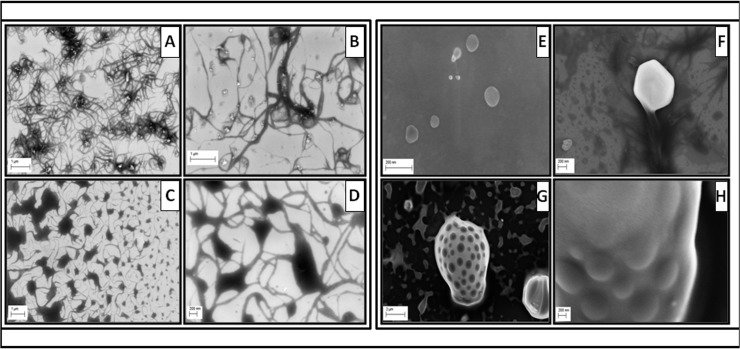
SEM images. Representative SEM micrographs of CNTs (panels a & b, scale 1 μm) and CNT-Indolicidin conjugates (panels c [scale 1μm] & d[scale 200 nm]) as well as GNPs (panles e & f, scale 200 nm) and GNP-Indolicidin conjugates (panels g [scale 2 μm] & h [scale 200 nm]) are shown.

Indolicidin (I) was conjugated with CNT and/or GNP covalently using EDC-NHS chemistry as described elsewhere [23]. Conjugated CNT with I (CNT_I) and GNP with I (GNP_I) were characterized by various optical spectroscopy [UV-Vis, fluorescence & Fourier Transformed Infra-Red (FT-IR)], Scanning Electron Microscopy (SEM) and IsoThermal Calorimetry (ITC). Results from UV-Vis and fluorescence spectroscopy is shown in Fig 2A–2C. Association constants and stoichiometry of binding were determined by Scatchard plots (Fig 2D–2G). Comparative FT-IR (Fig 3A and 3B) spectral analysis confirmed conjugation of Indolicidin to CNT and GNP followed by validation of the conjugation by thermodynamic analysis (Table 1) by ITC. We also compared SEM images (Fig 4A–4H) for further confirmation and characterization.

**Table 1:** Thermodynamic parameters of Indolicidin binding to CNTs and GNPs.

	ΔH ±S.D. (kcal/mole)	ΔS ±S.D. (cal/mol/degree)	ΔG ±S.D. (kcal/mole)
CNT-Indolicidin	-3200.0 ± 267.7	-11000.0 ± 1435.2	-2903.0 ± 254.6
CNT+Indolicidin	-100.0 ± 13.2	-355.0 ± 46.6	-90.5 ± 14.7
GNP-Indolicidin	-4300.0 ± 389.2	-14000.0 ± 1604.9	-3922.0 ± 371.8
GNP+Indolicidin	-2800.0 ± 236.4	-9300.0 ± 877.3	-2548.9 ± 233.6

We observed (Fig 2A) approximately 2-fold and 5-fold increase in absorbance upon covalent conjugation of indolicidin with CNT and GNP, respectively. Covalent conjugation quenched fluorescence of indolcidin in both cases but it was quenched significantly more for CNT than GNP (Fig 2B and 2C). We observed a blue shift of 18 nm in the fluorescence emission spectrum of GNP-conjugated indolicidin, however this phenomenon was not observed in case of CNT conjugation of the peptide., Fluorescence lifetime values of free and conjugated indolicidin were determined and did not see any significant difference between free and conjugated indolicidin (Table 2). The unchanged lifetime indicated ground state quenching of free fluorescence quenching and also revealed that peptide remains unperturbed in terms of conformation. We further established the strength and stoichiometry of binding and the energy associated with the binding. Fraction bound with increasing concentration of the peptides as determined by spectrophotometric titration for association of indolicidin with CNT (Fig 2D) and GNP (Fig 2F). Values of association constant (K) and stoichiometry (n) of binding, determined by Scatchard plot, are shown in Fig 2E (CNT_I) and 2G (GNP_I). Results indicated that GNP has two different binding sites (high and low) for the peptide while CNT has one. There was no significant association observed when non-activated CNT or GNP was mixed with indolicidin (spiked samples, CNT-I or GNP-I). This observation was further supported by thermodynamic parameters (Table 1).

**Table 2:** Fluorescence Lifetime values in nanoseconds (ns) for various indolicidin formulations with nanoparticles.

Sample Id	τ_1_(ns)	τ_2_(ns)	χ^2^	τ_F_ *(ns)
Indolicidin	0.86(±0.06)	2.70(±0.44)	1.09	1.38(±0.12)
CNT-Indolicidin	0.84(±0.04)	2.31(±0.32)	1.16	1.32(±0.16)
GNP-Indolicidin	0.82(±0.09)	2.74(±0.13)	1.42	1.36(±0.14)
CNT+Indolicidin	0.92(±0.11)	2.57(±0.67)	1.12	1.48(±0.11)
GNP+Indolicidin	0.89(±0.07)	2.45(±0.73)	1.33	1.44(±0.18)

τ_F indicates average of two (_τ_1 &_ τ_2) lifetime values._

Fourier Transformed Infrared (FT-IR) spectroscopy confirmed that indolicidin is covalently conjugated with CNT and GNP. Fig 3A and 3B reveal that the C-O stretch at 1085 cm^-1^ of the COOH groups of indolicidin. Fig 3A reveals that this band at 1085 cm^-1^ splits into multiple bands at 1051 cm^-1^ and 974 cm^-1^ suggesting amide C-N stretch for CNT_I conjugates. Two other new spectral bands appear at 1120 cm^-1^ and 1226 cm^-1^ representing the C-O stretch. In addition, presence of new bands at 1524 cm^-1^ and 1330 cm^-1^, corresponding to N-H in-plane and C-N bond stretching, respectively, further confirms the presence of a amide linkage (Fig 3A). The characteristic C-O stretching peak at 1085 cm^-1^ is fragmented into bands at, 1040 cm^-1^ and 980 cm^-1^ for GNP_I conjugate. The split peaks at 1040 cm^-1^ and 980 cm^-1^ represent C-N stretch present in an amide bond. The characteristic spectra of the free nanomaterials and the non-conjugated nanomaterials and peptide dispersions were compared with those of the conjugates where the conjugates showed remarkable band shifts and occurrence of newer bands indicating a successful conjugation reaction.

SEM images of free CNT, GNP and indolicidin conjugated CNT and GNP were further collected to image the difference between free nanomaterials (CNT and GNP) against the conjugated CNT and GNP (Fig 4).

**Fig 5:**
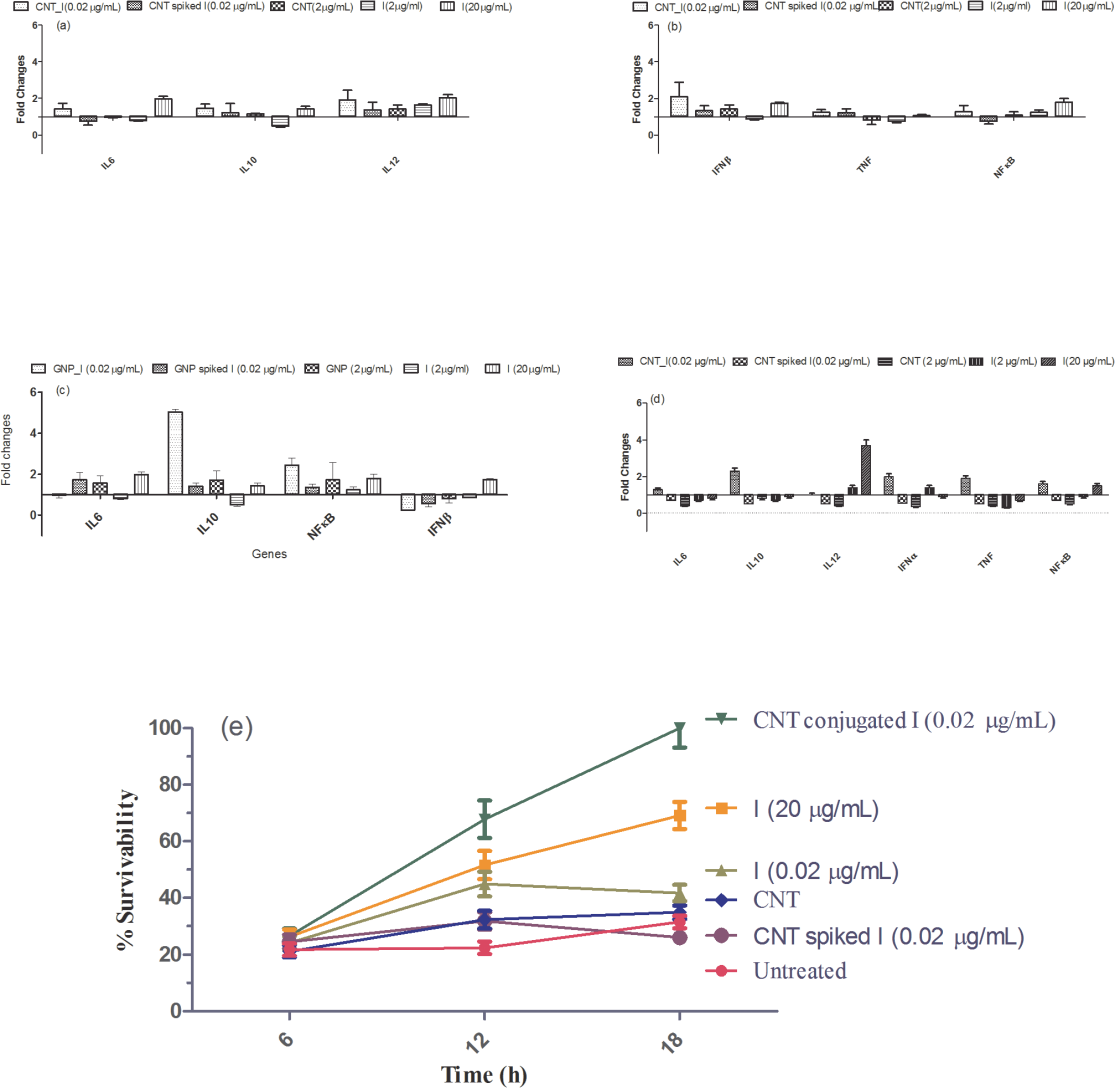
Transcriptional gene expression values and protection assay. Fold change values of representative gene expression at transcriptional level for various treatment conditions with respect to time matched untreated (a-c) RAW 264.7 and (d) THP-1 cells are shown. Gene labels are shown in X-axis and the conditions for each treatment are also shown in the figure. (e) Percent survivability of host cells under various treatment conditions with respect to time matched untreated samples of host cells at various time points post challenge is shown. Symbols for treatment conditions are shown in the figure. Methodology to determine percent survivability is shown in the text.

After characterizing indolicidin conjugated CNT and GNP, we checked for short term toxicity of the conjugates compared to free nanomaterials and other controls by measuring the cell viability. Dosage of free indolicidin was decided from viability studies at 50, 20, 10, 2, 0.2 and 0.02 μg/mL of the peptide using murine macrophage RAW 264.7 cells. Based on optimal survival and response in activating select innate immune genes, such as IL-6, IL-10, IL-12, IFN, TNF and NFκB, we selected a standardized dose of free indolicidin, at 20 μg/ml. Dose, selected for the current study, was also matching with reported dosage *in vitro*[24]. Dosage lower than 10 μg/mL did not have significant effects on innate immune genes selected to study activation upon treatment (Fig 5A, 5B and 5C). We did similar titrations with conjugated indolicidin and observed that even at 0.02 μg/mL concentration of indolicidin when conjugated with either CNT or GNP it is highly potent in activating select innate immune genes. Fig 5A–5D) reveal that free CNT or GNP as well as spiked samples do not have any effects. It is also important to note that while CNT_I activates both pro- (IL-6, IL-12, IFN, TNF and NFκB) and anti-inflammatory (IL-10) genes GNP-I activates mainly IL-10 at indolicidin concentration of 0.02 μg/mL. Because CNT-peptide conjugates showed differential regulation of both pro- and anti-inflammatory genes in murine macrophage cells (RAW 264.7), we further tested effects of CNT and peptide-conjugates in PMA treated differentiated and adherent human monocytes THP-1 (Fig 5D). It was observed that CNT conjugates showed similar behavior in adherent THP-1 cells as well.

We tested for ability of protection of indolicidin conjugated with CNT and GNP in murine macrophage cells RAW 264.7 and in Thp-1 cells. Both RAW 264.7 and ThP-1 cells were treated for 6h individually with free indolicidin, CNT, CNT_I and CNT spiked with indolicidin. Time matched untreated cells were used as control. All treated and untreated cells were challenged by *Salmonella typhimurium* serovar enterica. Relative survivability was estimated from viability of host cells at 6, 12 and 18 h post *Salmonella* challenge with respect to viability of time matched untreated cells. It was observed that CNT_I at 0.02 μg/mL was able to protect either type of cells significantly better than cells treated with free indolicidin at 20 μg/mL and other control treatment conditions (Fig 5E).

## Discussion

We report here conjugation of indolicidin with CNT and GNP and their characterization to establish the fact that the peptide is covalently conjugated. Significant hyperchromicity was observed in both (CNT and GNP) cases of conjugates as compared to free Indolicidin and the free nanomaterials (CNT and GNP). We also used controls by spiking free CNTs and GNPs with free indolicidin to check if hyperchromicity is due to conjugation. Spiked samples did not yield any significant changes in the absorbance values of indolicidin. Blueshift observed upon cojugation of indolicidin with GNP indicates more hydrophobic or buried environment of tryptophans present in indolicidin. This observation is further verified by other methodologies presented.

Comparing fluorescence intensity between the free peptide and its conjugates for both CNT and GNP, we observed that the fluorescence intensity obtained from CNT_Indolicidin conjugate was sixty times quenched than that of free Indolicidin. On the contrary, the fluorescence intensity of GNP_Indolicidin conjugate was only six times quenched. The spiked samples used as negative controls exhibited that fluorescence intensity of indolicidin is higher than the fluorescence intensities of CNT and GNP conjugates. The fluorescence intensities of free functionalized CNTs and GNPs were considered as baseline and therefore used for normalization of the spectra obtained from CNT_Indolicidin and GNP_Indolicidin conjugates as well as their non-conjugated counterparts. No significant differences were found between the fluorescence intensities of free CNTs and free GNPs. No significant changes, however, in fluorescence lifetimes were observed between free indolicidin and the conjugated peptide, implying that integrity of the peptide was conserved even upon conjugation.

Binding isotherm further characterizes the values of association constants and stoichiometry of binding. CNT binding is monophasic with stoichiometry of 1:1 while binding of the peptide with GNP was bi-phasic (perhaps two different binding sites with different affinities), but stoichiometry was 1:1 (GNP:peptide) for both binding.

FT-IR studies (Fig 3) revealed that a few specific split peaks (could be characteristic of binding) are formed as a result of conjugation of the peptide to the nanomaterials as compared to the controls where no such binding has taken place. FT-IR spectra of indolicidin spiked to CNT and GNP are somewhat similar to the spectra of free indolicidin, implying absence of any non-selective binding of indolicidin to CNT or GNP. Specific split peaks were, however, observed at the fingerprint region (around 1100 cm^-1^) of the spectra signifying formation of new bonds for CNT_ and GNP_Indolicidin conjugates.

Energetics of indolicidin conjugation to CNTs and GNPs were further characterized using isothermal calorimetry titrations to measure thermodynamic parameters such as enthalpy, entropy, and free energy associated with binding (Table 1). Both CNT and GNP conjugates, with indolicidin, were enthalpy driven. We also imaged the conjugates and controls by SEM (Fig 4). As expected, free CNTs appeared as thread-like extensions while the conjugates form more aggregate like structures. As the peptide is cationic in nature, electron beams get highly absorbed on the surface of the peptide structure thus yielding black aggregate-like structures in the CNT-conjugate. Similarly, in case of the GNP and GNP_Indolicidin, the conjugated peptide aggregating on the surface of the gold nanoparticles also appear as black spots due to the extensive absorption of the electrons from the electron beam due to the cationic nature of indolicidin. In case of nanoparticles with non-conjugated indolicidin, the peptide was localized surrounding the free CNTs and GNPs. Important to note that free gold nanoparticles display a brighter surface by deflecting the incident beam of electrons hence.

From gene expression profile data, we infer that free indolicidin, at a lower dosage, does not have any significant modulatory effect on the early innate immune system unlike its higher concentrations. Nonetheless, indolicidin in its lower concentration once conjugated to the nanoparticles are able to significantly increase gene expression of certain immune modulatory cytokines as compared to low concentrations of free peptide. Therefore, in order to minimize unwanted toxicity and the harmful side effects, indolicidin needs to be used at a lower therapeutic dosage; this is feasible by conjugating them to nanoparticles. In addition, it is also evident from the results that while CNT conjugated indolicidin is activating more pro-inflammatory genes, GNP conjugated indolicidin activates Il-10 a gene with anti-inflammatory function. As shown in Fig 5A–5C, free indolicidin, at a higher dose, yields a high expression of IFNβ which is an immune stimulatory cytokine. However, at a lower concentration, the free peptide has no significant effect unless conjugated to the CNTs. The CNT_Indolicidin conjugate significantly increases the expression fold change value almost to a similar extent to that of the higher dosage of indolicidin. Therefore, the CNTs have increased the efficacy of the peptide in such a way that it can be used at a minimal concentration, yet activating the innate immune system to an optimum degree. On the contrary, GNP_Indolicidin conjugate is found to down regulate the IFNβ expression as compared to free indolicidin. This demonstrates that the GNP conjugate follows an immune-suppressing mechanism. In addition, the remarkable increase of IL-10 upon treatment with the GNP-Indolicidin conjugate (Fig 5C), further confirms its immune suppressive property. IL-10 expression is increased four times by the GNP_Indolicidin conjugate as compared to the free peptide and its CNT conjugated equivalent. Indolicidin generally imparts a stimulating effect on the innate immune system, but conjugation to GNPs modulates them to produce an immune suppressive effect. The CNT-Indolicidin conjugate in this case elicits a similar expression profile as the free indolicidin at a higher concentration. Fig 5 further reveals that the free peptide at a lower concentration (0.02 μg/mL) does not elicit any significant innate immune response, however when conjugated to CNTs and GNPs, it elicits a significant immune modulatory response at 0.02 μg/mL.

Challenge study with *S*. *typhimurium* inferred that upto 6h post infection, RAW264.7 or ThP-1 survivability was unaffected in all conditions. However, at 12h and 18h we observed that CNT conjugated indolicidin at 0.02 μg/mL protected the cell from challenge of the bacteria significantly better than free indolicidin at 20 μg/mL.

## Conclusion

The aim of the study was to increase efficacy of indolicidin by conjugating these host defence peptides to different classes of nanomaterials. As portrayed by the physical characterization experiments, we could conjugate indolicidin to both CNTs and GNPs. The binding of the indolicidin to the nanomaterials was successfully characterized by various techniques. Conclusive evidence of the conjugation was obtained from the FT-IR experiments. The peaks obtained in the fingerprint region of the FT-IR spectra of the CNT-Indolicidin and GNP-Indolicidin conjugates indicated additional C-N bond vibrational frequencies corresponding to the new covalent bond that formed between indolicidin and the nanomaterials. As compared to the free peptide spectra, the emergence of new peaks in the fingerprint region successfully confirmed the conjugation reaction.

Results from qRT-PCR experiments revealed that, CNT-Indolicidin and GNP-Indolicidin conjugates induced complemetary innate immune gene activation. CNT-indolicidin could also protect host cells against bacterial protection significantly better than free indolicidin at 1000-fold less concentration.

There are only a few reports suggesting effective drug delivery by conjugating with CNT and/or GNP [25–29]. It was shown that single walled CNTs coated with human serum albumin or polyethylene glycol could activate various complement pathways in cells[25]. Another report has delivered ssRNA using gold nanorod to a lung epithelial cell line to show activation of innate immune response to inhibit influenza virus infection *in vitro* [26]. Besides, there are a few studies which revealed bio-distribution and delivery efficiency with CNT and GNP in cells[27–29]. Current report along the line has further shown that CNT and GNP can also be very effective in increasing efficacy of a drug significantly and the peptide conjugated with nanoparticle at 1000-fold less concentration than free peptide maintained similar innate immune activation and inhibition efficacy against bacterial chellenge. Current results could now be expanded to study efficacy of the conjugates against key viral infection and to be tested in animal models.

## Supporting Information

S1 Table Primer sequences used for qRT-PCR gene expression assays. (DOC)
