# Supplementary material for: Immune Activation Efficacy of Indolicidin Is Enhanced upon Conjugation with Carbon Nanotubes and Gold Nanoparticles
Source: PLoS One. 2015 Apr 15;10(4):e0123905. doi: 10.1371/journal.pone.0123905 (PMC4398554; doi:10.1371/journal.pone.0123905)
Supplement: S1 Table — (DOC) [file pone.0123905.s001.doc]

**Supporting Information**

**Title:** Immune activation efficacy of Indolicidin is enhanced upon conjugation with Carbon nanotubes and gold nanopartcles

Abhinav Sur, Biswaranjan Pradhan, Arka Banerjee, Palok Aich^*^

**Table 1**s: Primer sequences used for qRT-PCR gene expression assays

| Target Gene | Primer | Primer Sequence | Bases | Accession No. |
| --- | --- | --- | --- | --- |
| IL-6 | IL6(F) | AGACAAAGCCAGAGTCCTTCAGAG | 24 | NM_031168.1 |
|  | IL6(R) | CCACAGTGAGGAATGTCCACAAC | 24 | NM_031168.1 |
| IL-12 | IL12(F) | CGCCCAAGAACTTGCAGATGAAGC | 24 | NM_008352.2 |
|  | IL12(R) | CGCCTTTGCATTGGACTTCGGTAG | 24 | NM_008352.2 |
| IL-10 | IL10(F) | AGGCAGTGGAGCAGGTGAAGAGTG | 24 | NM_010548.2 |
|  | IL10(R) | GCTCTCAAGTGTGGCCAGCCTTAG | 24 | NM_010548.2 |
| TNF | TNF(F) | CCACGTCGTAGCAAACCACCAAG | 24 | NM_013693.2 |
|  | TNF(R) | TGCCCGGACTCCGCAAAGTCTAAG | 24 | NM_013693.2 |
| IFNβ | IFNβ(F) | AACTCCACCAGCAGACAGTGTTTC | 24 | NM_010510.1 |
|  | IFNβ(R) | TCCGCCTCTGATGCTTAAAGGTTG | 24 | NM_010510.1 |
| NFKβ | NFKβ(F) | CAGGGTATGGCTACTCGAACTACG | 24 | NM_008689.2 |
|  | NFKβ(R) | CCAGATGTGACTTCCAGCAGATCC | 24 | NM_008689.2 |
| β-Actin | β-Actin (F) | CTGAGCGCCAGGTCATCACTATTG | 24 | NM_001101.3 |
|  | β-Actin (R) | GACAGCACTGTGTTGGCATAGAGG | 24 | NM_001101.3 |
